# Repeated failure of implants at the same site: a retrospective clinical study

**DOI:** 10.1186/s40902-019-0209-1

**Published:** 2019-07-10

**Authors:** Dong-Woo Kang, So-Hyun Kim, Yong-Hoon Choi, Young-Kyun Kim

**Affiliations:** 1 Department of Oral and Maxillofacial Surgery, Section of Dentistry, Seoul National University Bundang Hospital, 300, Gumi-dong, Bundang-gu, Seongnam-si, Gyeonggi-do 463-707 Korea; 2 Department of Orthodontics, Section of Dentistry, Seoul National University Bundang Hospital, Seongnam, Korea; 3 Department of Conservative Dentistry, Section of Dentistry, Seoul National University Bundang Hospital, Seongnam, Korea; 4 Department of Dentistry & Dental Research Institute, School of Dentistry, Seoul National University, Seoul, Korea

**Keywords:** Implant failure, Cluster failure, Repeated failure

## Abstract

**Background:**

Implants are becoming the first choice of rehabilitation for tooth loss. Even though they have a high success rate, failures still occur for many reasons. The objective of this study is to analyze the reasons for recurring failure at the same site and the results of re-implantation.

**Methods:**

Thirteen patients (11 males and 2 females, mean age 60 ± 9.9 years) who experienced implant surgery failure at the same site (same tooth extraction area) two or more times in the Department of Oral and Maxillofacial Surgery, Seoul National University Bundang Hospital, between 2004 and 2017 were selected. The medical records on a type, sites, diameter, and length of implants; time and estimated cause of failure; and radiographs were reviewed. Data were collected and analyzed retrospectively, and the current statuses were evaluated.

**Results:**

A total of 14 implants experienced failure in the same site more than two times. Twelve implants were placed in the maxilla, while 2 implants were placed in the mandible. The maxillary molar area was the most common site of failure (57.1%), followed by the mandibular molar, anterior maxilla, and premolar areas (14.3% each).

The first failure occurred most commonly after prosthetic treatment (35.7%) with an average period of failure of 3.8 months after loading. Ten cases were treated as immediate re-implantation, while the other 4 were delayed re-implantation after an average of 3.9 months. The second failure occurred most commonly after prosthetic treatment (42.9%), with an average of 31 months after loading; during the healing period (42.9%); and during the ongoing prosthetic period (14.3%). In 3 cases (21.4%), the treatment plan was altered to an implant bridge, while the other 11 cases underwent another implant placement procedure (78.6%).

Finally, a total of 9 implants (64.3%) survived, with an average functioning period of 60 months.

**Conclusions:**

Implants can fail repeatedly at the same site due to overloading, infection, and other unspecified reasons. The age and sex of the patient and the location of implant placement seem to be associated with recurring failure. Type of implant, bone augmentation, and bone materials used are less relevant.

**Electronic supplementary material:**

The online version of this article (10.1186/s40902-019-0209-1) contains supplementary material, which is available to authorized users.

## Background

Treatment for rehabilitating partially edentulous or fully edentulous areas with implants has established itself as a universal and predictable dental treatment option [1–5]. Although the rate of implant success is increasing with technology advancements, implant failures have been continuously reported in a small number of patients. Although the cause is not clearly identified for all patients, it is possible that implant failure is repetitive and results in concentrated cluster behavior [6, 7]. A cluster is defined as more than one implant failure per patient and does not necessarily have to occur in the same position or within the same quadrant [7]. It is assumed that patients who fail intensively exhibit special characteristics.

The main causes of implant failure are systemic disease, poor oral hygiene, irradiation in the area of the head and neck, chronic periodontitis, lack of experience of the clinician, poor bone quality or bone quantity, implantation in the maxilla, implantation in the molar area, excessive smoking, use of short-length implants, lack of initial stability, immediate loading or early loading with poor initial stability, inadequate implant design, and excessive number of implants. However, it is often difficult to determine the cause [8–11].

Failure of implants can be divided into early implant failure occurring during osseointegration or during the beginning of loading and delayed implant failure occurring after osseointegration and completion of prosthetic treatment. Early implant failures are caused by failure of initial osseointegration between the implant surface and the surrounding bone by the change of the treatment plan. The main causative factors are contamination, infection, peri-implantitis, trauma during or after surgery, inadequate healing, and early loading. On the other hand, delayed implant failures are assumed to be due to overloading, trauma, excessive bite force caused by parafunction, and pathological processes caused by infection [12, 13].

The purpose of this study is to seek a highly predictable implant therapy option by evaluating a retrospective clinical study on repeated implant failure in the same site to analyze the estimated causes and to determine appropriate and effective treatment methods in the event of repeated implant failure.

## Main text

### Materials and methods

This study was conducted under the approval of the Bioethics Review Committee at Seoul National University Bundang Hospital (IRB: B-1901-514-103). A total of 14 implants placed by a single surgeon and 12 patients whose implants failed at least twice in the same anatomical area were studied from January 2004 to May 2017 in the Oral and Maxillofacial Surgery Department of Seoul National University Bundang Hospital. Ten men and two women with an average age of 61.4 ± 8.9 years were included in the study. Implant failure in this study comprised loss due to osseointegration failure, prosthetic complications, or peri-implantitis and excluded surviving implants with severe marginal bone loss. Failures were classified into three categories: during the healing period, during prosthetic treatment, and after completion of prosthetic treatment.

Patient age, sex, underlying disease, cause of tooth loss or extraction (such as periodontitis, trauma, fracture, dental caries, congenitally missing, and severe infection), implant location, type of implant, implant diameter and length, surgery method, additional bone grafting, assumed cause of failure, time of failure, post-failure treatment method, type of prosthesis, and progress at the final observation were evaluated retrospectively with clinical records.

The location of implantation was divided into the maxilla and mandible, followed by anterior, premolar, and molar regions. The time of failure was divided into during the healing period, during prosthetic treatment, and after completion of prosthetic treatment. The estimated causes of failure were identified by clinical and radiological findings and intra-oral examinations and included attrition, bruxism, and clenching. Indications of parafunction were based on the contents of the International Classification of Sleep Disorders [14]. The methods of treatment were divided into sleeping, immediate re-implantation, delayed re-implantation, and no implantation. The final progress of the implant was confirmed by the presence of complications or unusual findings at the last observation.

## Results

The first 14 implants placed in 12 patients failed repeatedly along the same time frame. Ten male patients (83.3 percent) and 2 female patients (16.7 percent) were evaluated in the study. Twelve repeatedly failed implants occurred in the maxilla and 2 in the mandible. In 2 patients, 2 implants at other sites each failed repeatedly. The distribution of failure by position was largest at 57.1% for the maxillary molar area (Table 1). Four of the male patients smoked for an extended period of time, and oral parafunctional habits such as bruxism and clenching were observed in 7 male patients, including repeated intake of hard or tough foods.

**Table 1:** Patients and materials

	Age	Position	1st implant	2nd implant	3rd implant
M1	74	#11	TiUnite (4×11.5)	Osstem US II	–
M2	43	#46	Superline (5×8)	Superline (7×8)	Superline (5×10)
M3	68	#46	Superline (4.8×12)	SinusQuick IS (5×11.5)	Superline (7×10)
M4	61	#26	Osstem US III (5×11.5)	Osstem US III (4×11.5)	Osstem TS III BIOSA (5×11.5)
M5	62	#25	3-I Osseotite NT (5×13)	3-I Osseotite NT (5×15)	Osstem US II (5×18)
M5	62	#26	3-I Osseotite NT (5×11.5)	3-I Osseotite NT (6×13)	Osstem US II (5×18)
F1	66	#11	3-I Osseotite External (5×11.5)	Osstem US II (4×13)	–
M6	57	#26	3-I EB (5×14)	Superline (7×8)	Superline (4.5×8)
M7	70	#26	Superline (5×14)	Superline (7×10)	Superline (7×12)
M8	64	#26	3I Osseotite certain	Osstem US II (5×15)	–
M9	63	#26	Osstem TS III HA (5×10)	Superline (4.8×12)	Osstem TS IV (5×11.5)
M9	63	#27	Osstem TS III HA (5×10)	Superline (5×15)	Osstem TS IV (4.5×10)-
F2	62	#27	3-I NanoTite external (5×10)	Superline (6×10)	Superline (5×10)
M10	47	#15	Superline (4.3×10)	Superline (4.8×14)	Superline (4.8×12)

*M* male, *F* female

M5 and M9 repeatedly failed at two sites each

The initial implant lengths were 8 mm (1), 10 mm (4), 11.5 mm (6), 12 mm (1), 13 mm (1), and 14 mm (1). The implant diameters were 4 mm (2), and 5 mm (12). The implants that failed after initial placement were submerged beneath the crest of bone in 11 cases and non-submerged in 3 cases, accompanied by bone grafting in 8 cases. Bone grafting was performed by guided bone regeneration (GBR) and sinus bone grafting. The bone graft materials were manufactured by Auto BT® (Korea Tooth Bank Co., Seoul, Korea), BioOss® (Geistlich Pharma AG, Wolhausen, Switzerland), Inducera® (Osscotec, Cheonan, Korea), Orthoblast II® (Isotis Orthobiologics US, Irvine, CA, USA), and Exfuse^TM^ (Hanmi Co., Seoul, Korea). In 3 cases, barrier membranes were used, 2 of which were Bio-arm® (ACE Surgical Supply Company Inc., Brockton, USA) and 1 Ossix® plus (Datum Dental Ltd., Telrad, Israel). The failure period was after prosthetic treatment in 5 patients and during the healing period after implantation in 5 patients. After the prosthetic treatment was completed, implants failed after loading for an average of 3.8 months. Four implants failed during prosthetic treatment. The estimated causes of failure for 5 implants were overloading from oral parafunctional habits such as bruxism, clenching, or immediate loading; 3 from infections; and 6 from unknown causes (Table 2). The treatment of 10 first failed implants was replacement immediately after removal, while 4 underwent delayed replacement after an average of 3.9 months of healing (Table 3).

**Table 2:** Estimated cause of failure

Estimated cause of failure	1st failure	2nd failure	3rd failure
Overloading	5	3	–
Infection	3	1	1
Lack of secondary stability	–	3	–
Unknown	6	7	–
Total	14	14	1

**Table 3:** Treatment of failed implants

Treatment	1st failure	2nd failure	3rd failure
Immediate re-implantation	10	6	–
Delayed re-implantation	4	5	–
Removal of implant	–	2	1
Sleeping	–	1	1
Total	14	14	2

The secondary placed implant lengths were 8 mm (2), 10 mm (2), 10.5 mm (2), 12 mm (1), 13 mm (3), 14 mm (1), and 15 mm (3). The implant diameters were 4 mm (2), 5 mm (7), 6 mm (2), and 7 mm (3). Eleven implants were placed beneath the crest of bone, while 3 were non-submerged. GBR was performed in 5 implants with surrounding defects. The bone graft materials used were autogenous bone particles collected during the drilling procedure, ICB Cortical® (Rocky Mountain Tissue Bank, Aurora, CO, USA), Inducera® (Osscotec, Cheonan, Korea), DBX® (DePuy Synthes, Zuchwil, Switzerland), and BioOss® (Geistlich Pharma AG, Wolhausen, Switzerland), with an Ossix® plus (Datum Dental Ltd., Telrad, Israel) membrane used in 1 case. Six secondary implants failed after completion of prosthetic treatment and 6 failed during the healing period. Failed cases after completion of prosthetic treatment underwent an average of 31 months of loading. During prosthetic therapy, 2 implants were removed. The estimated causes of failure were overloading (3), insufficient secondary stability (3), infection (1), and unknown causes (7) (Table 2). The treatment for secondary implant failure was immediate replacement (6), delayed replacement (5) with an average of 3.8 months of healing, removal and no replacement (2), and 1 of sleeping beneath the alveolar bone for alteration of treatment to an implant bridge (1) (Table 3).

The widths of the third placed implants was 4.5 mm (2), 5 mm (7), and 7 mm (2), with lengths of 8 mm (1), 10 mm (4), 11.5 mm (2), 12 mm (2), and 18 mm (2). Five implants were submerged during placement, while 6 were non-submerged. One implant failed due to infection during the healing period, so re-implantation in that site was not performed, and an implant bridge was planned. Another implant was left within the alveolar bone due to insufficient osseointegration (Table 3).

Nine of the 14 implants that were replaced due to initial failure survived and underwent ideal loading, while 2 were monitored within the alveolar bone due to insufficient osseointegration or mobility. Three of the 14 implants were removed with an altered treatment plan, showing a final survival rate of 64.3%. The average marginal bone loss of the final implants was 0.21 ± 0.33 mm.

### Case reports

#### Case I

**Fig. 1:**
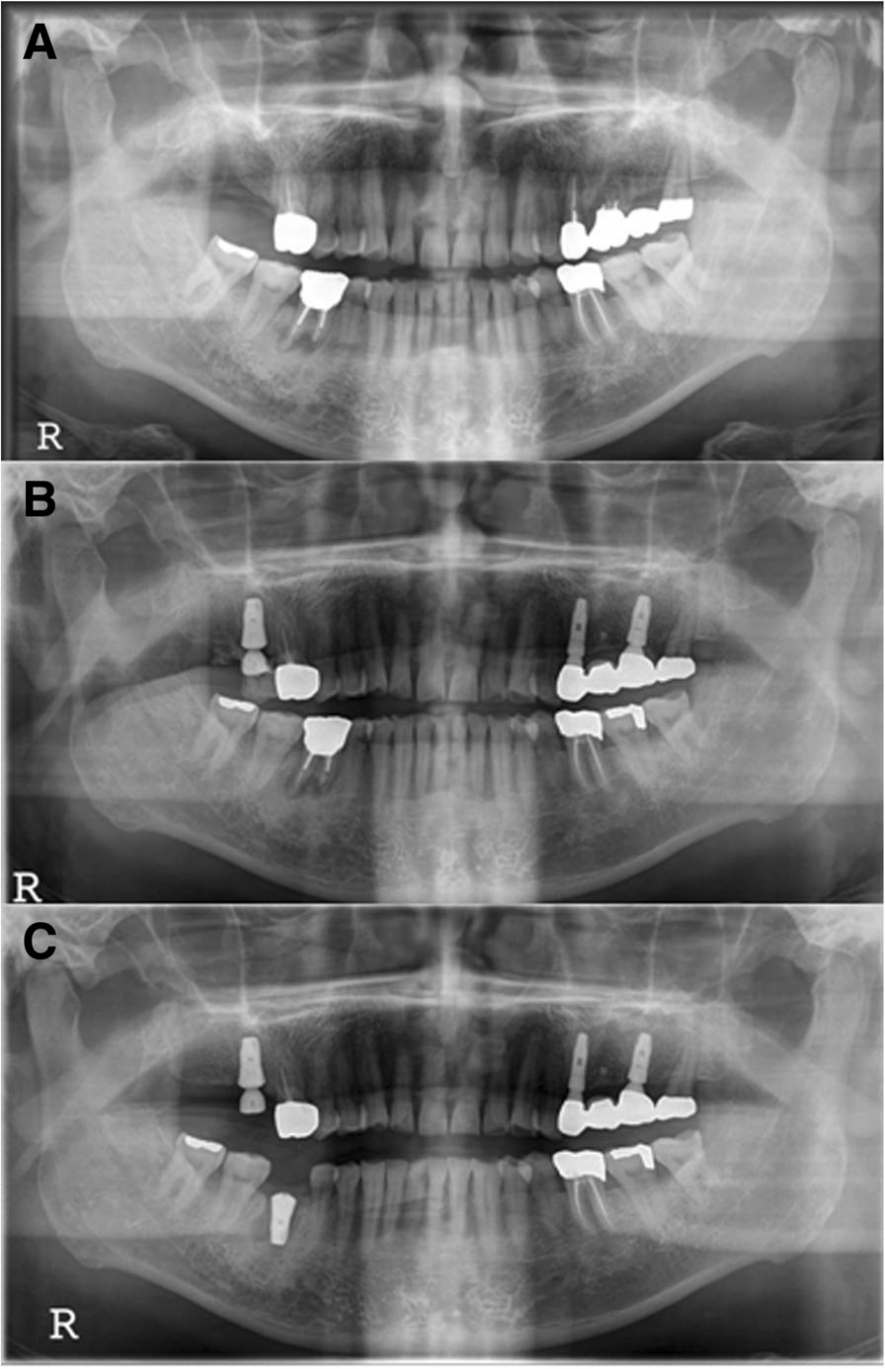
M3, panoramic radiograph, #46i surgery **a** initial panoramic radiograph, **b** pre-operative panoramic radiograph, **c** postoperative panoramic radiograph

**Fig. 2:**
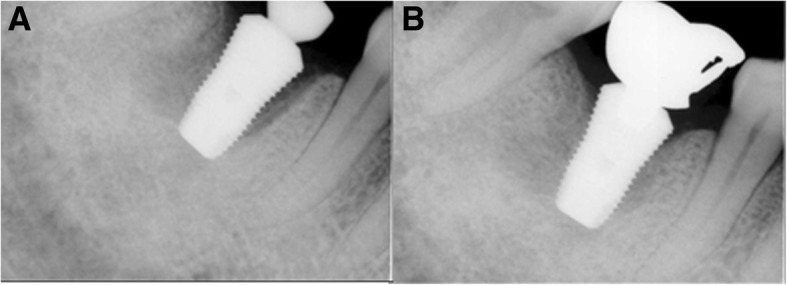
M3, periapical radiograph, **a** #46i second operation, **b** #46i prosthesis delivery

**Fig. 3:**
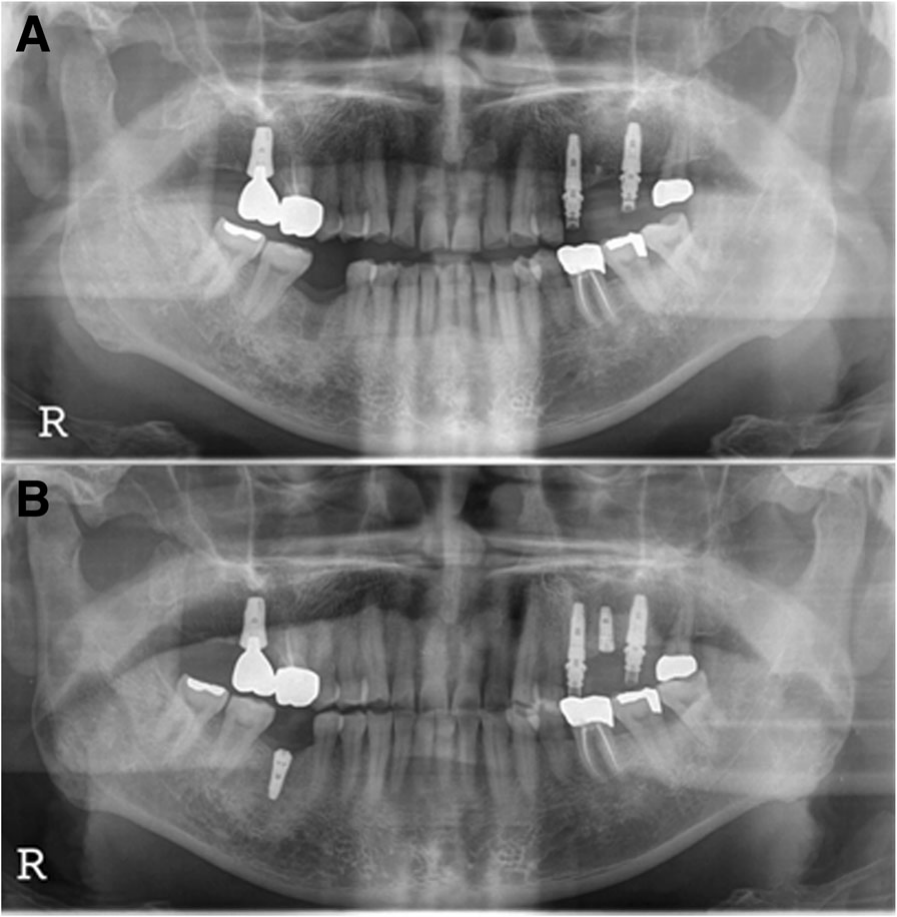
M3, panoramic radiograph, **a** postoperative 1-month removal of fixture state, **b** #46i, second installation

**Fig. 4:**
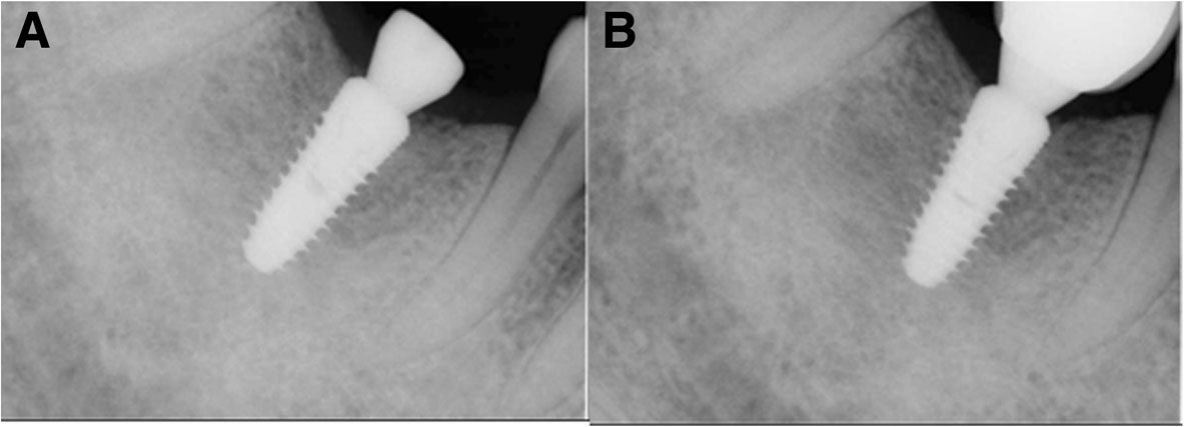
M3, periapical radiograph, **a** #46i, second operation, **b** #46i, prosthesis delivery

**Fig. 5:**
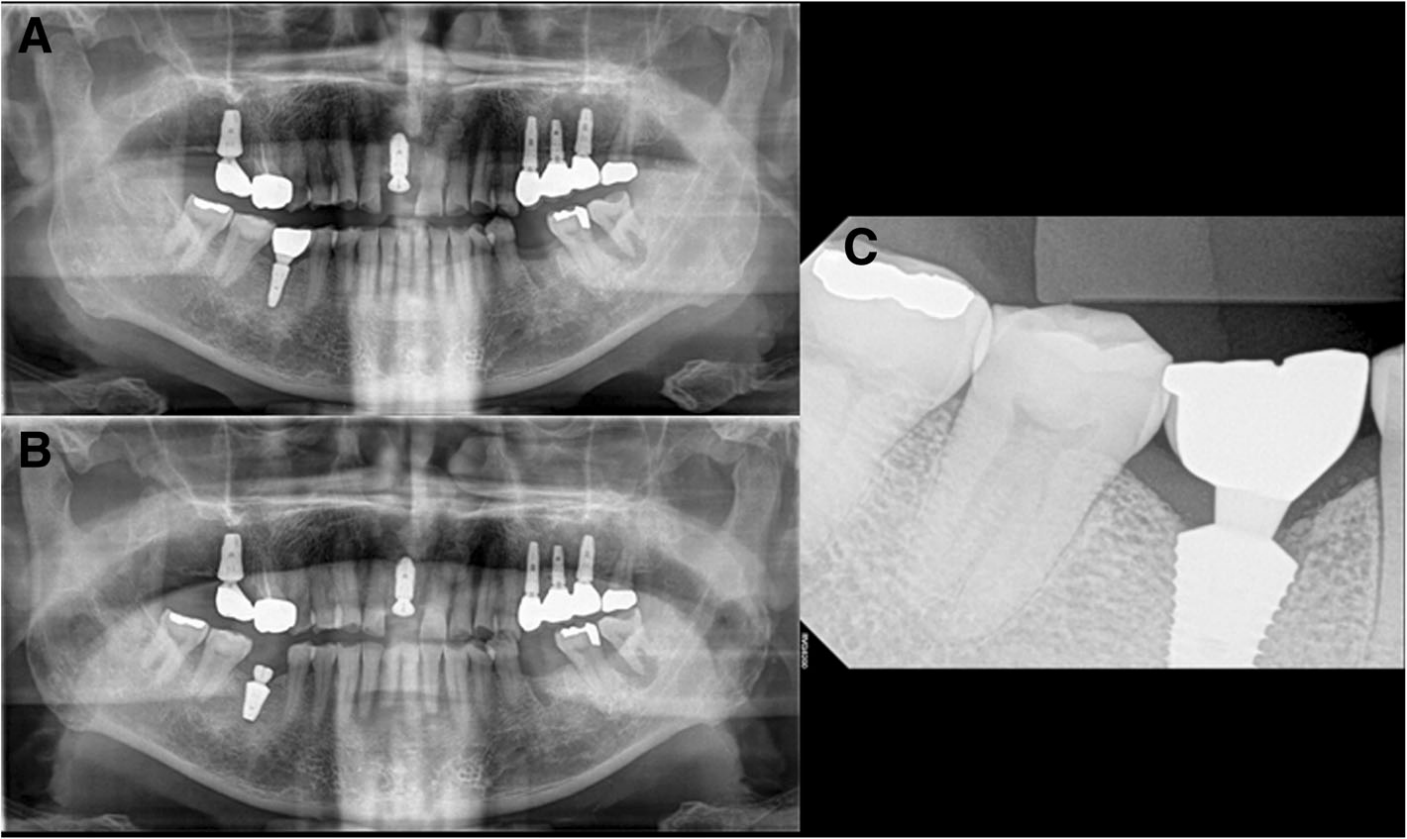
M3, radiographs **a** #46i second failure, preoperative panoramic radiograph, **b** #46i, third installation, **c** #46i, prosthesis delivery

A 68-year-old male patient (M3) with no underlying disease exhibited symptoms of #46 pain with a buccal gingival fistula. Tooth extraction and implant placement were planned under the diagnosis of a periapical abscess (Fig. 1a). After extraction of #46 in April 2008, an Implantium Superline 4.8 × 12 mm implant was placed. A buccally fenestrated 4-wall bony defect at the apical area was detected, and bone grafting was performed with Orthoblast II and a Bio-Arm barrier membrane with sutures (Fig. 1b, c). Four months later, the second surgery was performed (Fig. 2a). Six months after implantation, prosthetic treatment was carried out, and secondary stability was measured with an Osstell Mentor as 68 ISQ (Fig. 2b). Three months after insertion of the prosthesis, the patient exhibited symptoms of pain, hypersensitivity, and micromovement of the fixture. One month later, peri-implant curettage and antibiotic therapy were performed with an ISQ value of 59. Heavy occlusal forces with night clenching or bruxism were suspected due to fracture of the abutment connection of the #26 implant and observation of severe attrition on the upper and lower teeth. Eventually, the #46 implant was removed 6 months after prosthetic loading, and delayed re-implantation was planned for 3 months later (Fig. 3a). During the recovery period, a night guard was fitted to the upper dental arch to protect the teeth from parafunctional habits. In August 2009, a SinusQuick IS 5 × 11.5 mm implant was placed after the 3-month recovery period (Fig. 3b). At that time, the ISQ of primary stability was measured with an Osstell Mentor at 94. Three months later, the second surgery was performed, and the ISQ of secondary stability measured by the Osstell Mentor was 94 (Fig. 4a). Two months later, the final prosthetic treatment was completed (Fig. 4b). Since then, the implant has performed well, with regular check-ups 3 times a year. Two years later, night clenching and bruxism persisted with fracture of the cervical areas of residual teeth. Mobility of the #46 implant was observed in August 2016, approximately 6 years 7 months after prosthetic loading. After 2 months, the implant was replaced with a wide diameter Superline 7.0 × 10.0 mm implant with an ISQ of primary stability of 85, and the healing abutment was connected (Fig. 5a, b). Four months later, prosthetic treatment was completed (Fig. 5c). The final observation point was in November 2018, and the implant was maintained and had good prosthetic function after 1 year 9 months.

#### Case II

**Fig. 6:**
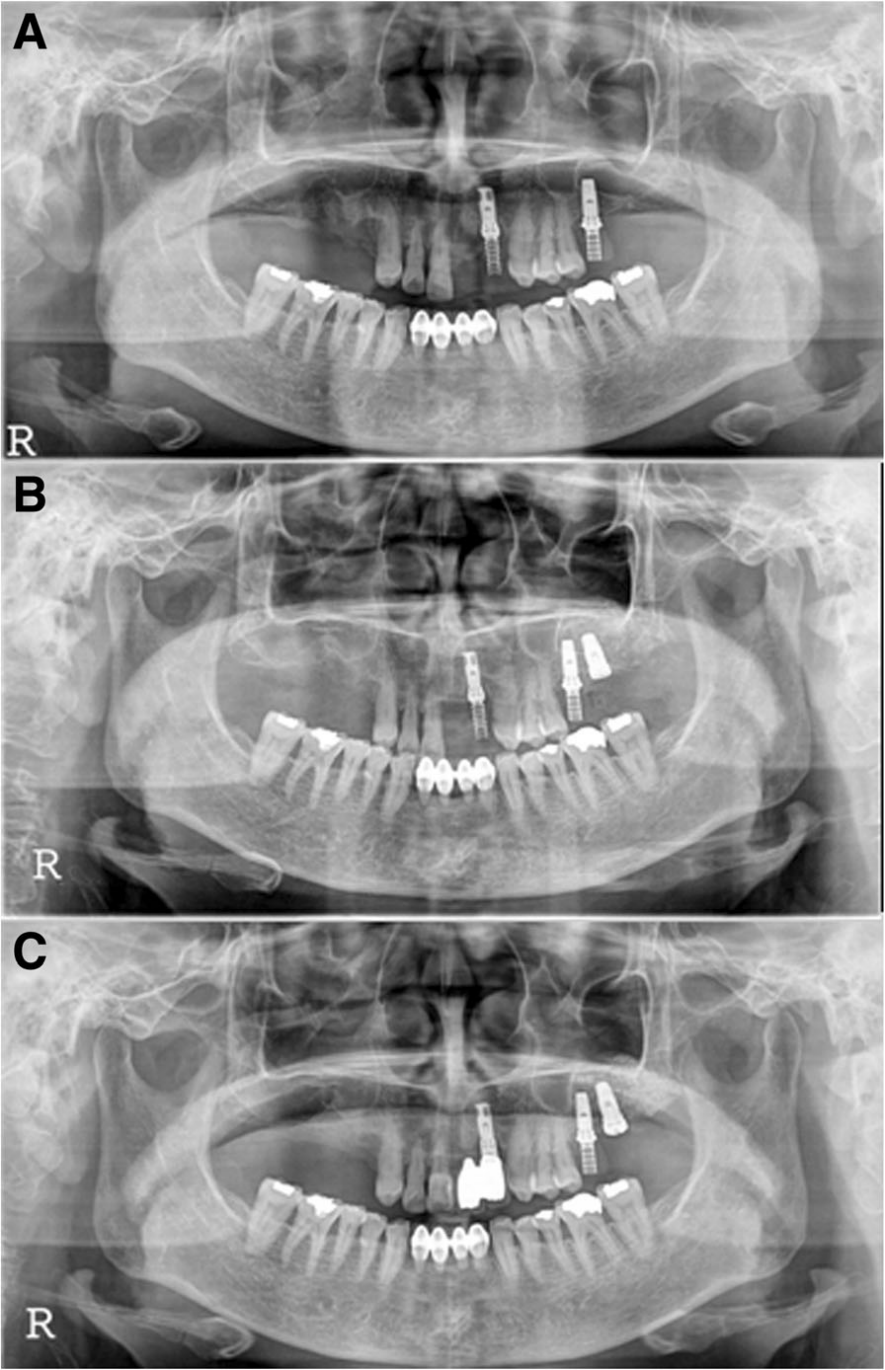
F2, Panoramic radiograph, #27i surgery **a** initial panoramic radiograph, **b** postoperative panoramic radiograph, **c** #27i second operation

**Fig. 7:**
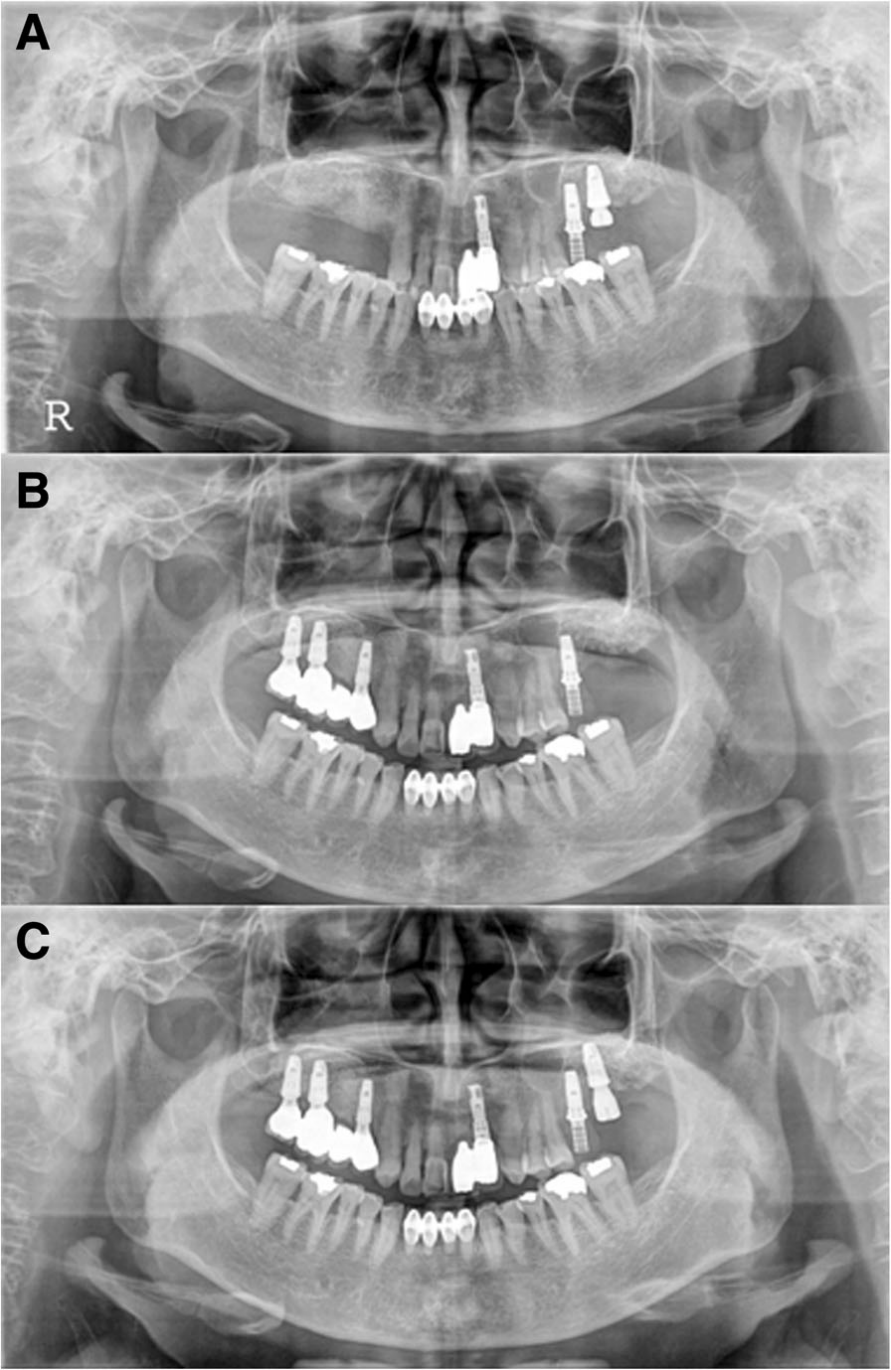
F2, panoramic radiograph, **a** #27i second installation, **b** #27i second failure, preoperative panoramic radiograph, **c** #27i, third installation

**Fig. 8:**
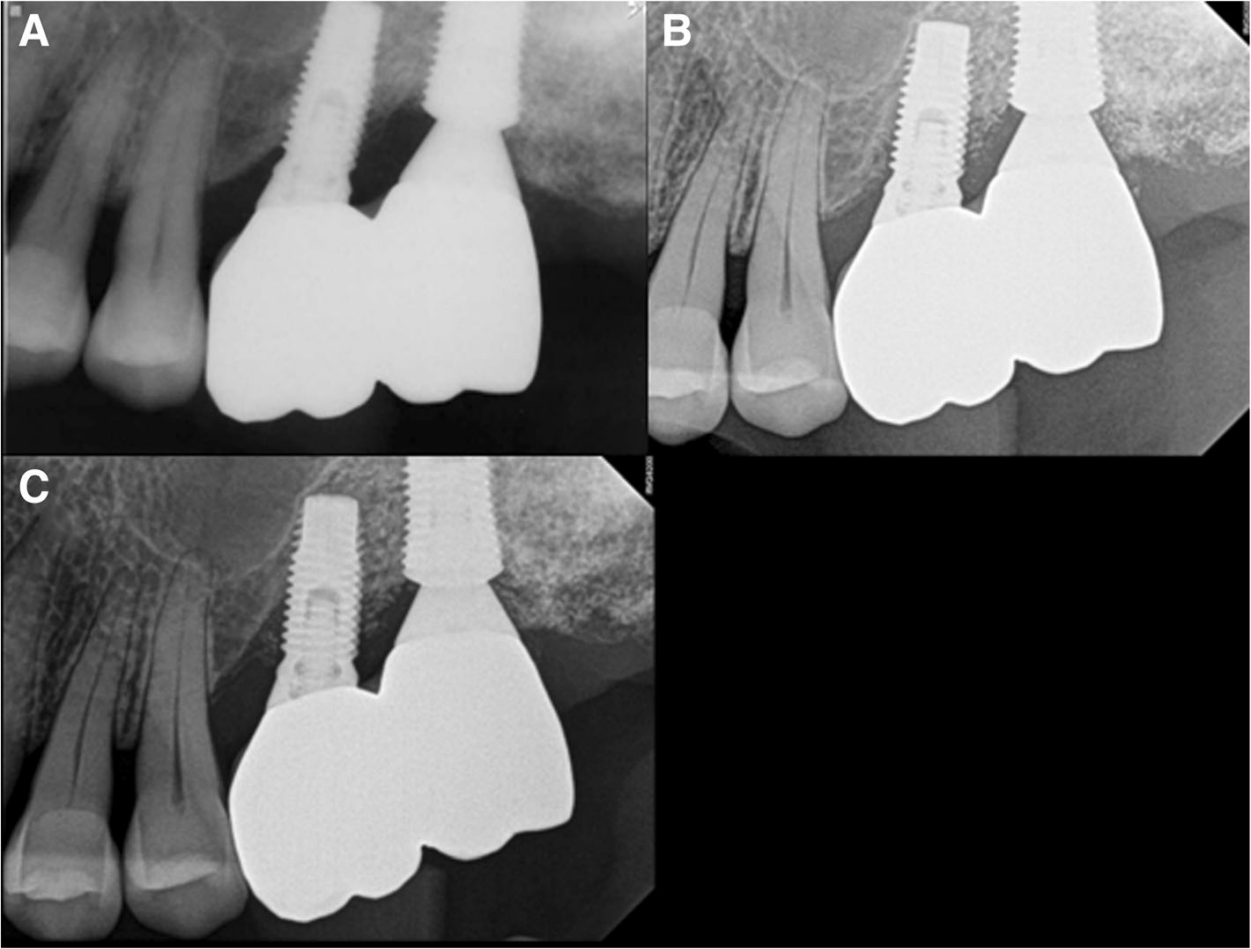
F2, periapical radiograph, **a** #27i, prosthesis delivery, **b** #27i peri-implantitis, #26i increasing radiolucent lesion, **c** #27i peri-implantitis, #26i mesial bone loss

**Fig. 9:**
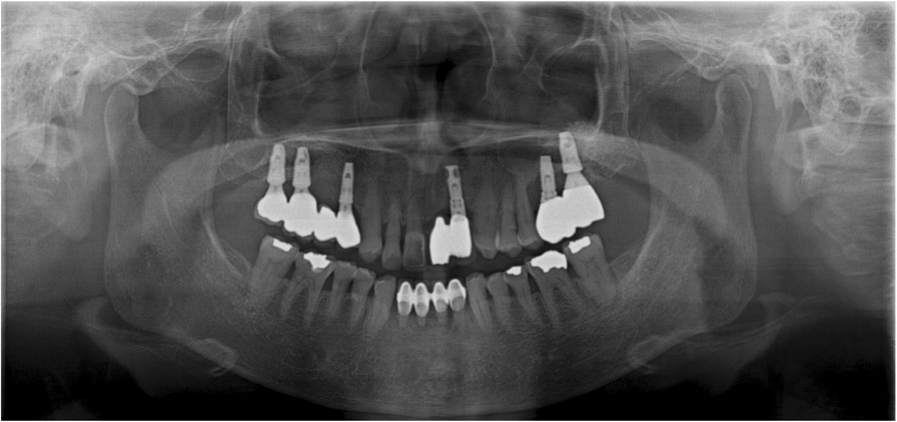
F2, the final panoramic radiograph showing bone loss at #26i and 27i

The F2 patient was 62 years old when she was first diagnosed and had no underlying disease. At the time of initial diagnosis in November 2011, she underwent extraction of #27 and bone grafting. Three years prior, maxillary sinusitis was reported at another dental clinic. The patient underwent endoscopic sinus surgery suspecting a tumor in the maxillary sinus at another otorhinolaryngology clinic and showed severe alveolar bone loss in both maxillary molar areas. An implant in the #26 site was placed at another dental clinic (Fig. 6a). In the CT view, the right maxillary sinus was observed to be close to the natural ostium, and an otorhinolaryngology consultation was obtained. In February 2012, bone grafting was performed by combining bone chips collected from the maxillary tuberosity with a bone rongeur, AlloMatrix, and InduCera after sinus lifting with a LASK (Lateral Approach Sinus Kit) kit by the lateral window approach. At the same time, supracrestal placement of implant #27 was performed with a 3-I NanoTite external 5 × 10 mm implant, and additional bone grafting was performed with Inducera and an AlloMatrix graft in the buccal area with an ISQ of primary stability measured with an Osstell Mentor at 66 (Fig. 6b). Three months later, the #27 implant's cover screw was exposed, the ISQ of secondary stability was measured with an Osstell Mentor at 62, and a healing abutment was connected (Fig. 6c). Five months after implantation, rotation of the implant fixture was detected during removal of the healing abutment for impression creation; treatment was stopped. One month later, the #27 implant was removed, and an Implantium Superline 6 × 10 mm was immediately placed using the trabecular compaction technique with ISQ of primary stability of 55 measured with an Osstell Mentor (Fig. 7a). Two weeks later, dull sounds and mild mobility of the fixture were observed. When checked 6 months after implantation, osseointegration was observed to have failed, and the implant was removed. After an 8-month healing period, the third implantation was performed with a flapless operation. A Superline 5 × 10 mm implant was placed, and the healing abutment was connected, with an ISQ of primary stability of 70 measured with an Osstell Mentor (Fig. 7b, c). Six months later, a cement-retained prosthesis was delivered (Fig. 8a). One year after prosthetic loading, peri-implantitis was observed. Therefore, subgingival curettage and injection of minocycline were performed in the gingival sulcus, and an increasing radiolucent lesion around the #26 implant was detected on periapical X-ray (Fig. 8b). After 3 months, peri-implant curettage on #26-27 implants, iBrush cleansing, KEY laser therapy, and injection of minocycline were performed. After 6 months, another round of curettage and minocycline injection was performed in the #26–27 implants (Fig. 8c). Marginal bone loss around the #26 and 27 implants was observed, but the implants survived without any symptoms. The final observation point was in January 2019, and the implants exhibited ideal loading at approximately 4 years 7 months (Fig. 9).

## Discussion

In this study, the most common area of repeated implant failure was the maxillary molars, with 12 sites in the maxilla and 2 in the mandible. This was similar to previous studies in which the failure rate was high in the maxilla and in molar areas [15, 16]. This is often attributed to the poor bone quality of the maxilla and the high degree of occlusal loading in molar areas.

Implant failure can be divided into early and late failures based on the time of failure. Early failure is failure to obtain osseointegration within several weeks or months after implant placement, mainly due to poor bone quality, necrosis of bone due to micro-trauma during surgery, bacterial infections around implants, lack of initial stability, immediate or early loading, smoking, or short-length implants [17, 18]. Delayed failure is destroyed osseointegration after functional loading and is thought to have a main cause of infection such as peri-implantitis or excessive overloading [19, 20]. Most of the failures of the first implants placed in this study occurred during the healing period or during the prosthetic period, while 5 cases with prosthetics failed early at an average of 3.8 months after loading. Studies by Zarb et al. and Naert et al. have reported a higher incidence of early failures than delayed failures and exhibit similar aspects to this study [21, 22].

The estimated causes of many primary failures in this study were overloading and infections, but causes were difficult to identify in 6 cases. In fact, 7 of the patients who experienced repeated implant delayed failure either began wearing previously fabricated appliances or newly produced night guards due to suspected oral parafunction, as seen in Case I. Suspected parafunctional habits include frequent eating of tough, hard foods such as squid and crabs; severe tooth attrition; bruxism or clenching during sleep; and cervical abfractions due to excessive clenching. Implant in Case I may also be considered as one of the causes of failure, as the insufficient depth of the implant placement in radiological analysis.

Assuming an unknown cause of failure in Case II, it is possible that the surface preparation of the 3-I NanoTite external type implant was insufficient or contaminated or that infection remained because the maxillary sinusitis was not fully controlled after sinus bone grafting in the other clinics. In addition, the bone graft may have been lost due to early exposure of the cover screw. The implant was placed supracrestally with surrounding bone grafting during the first surgery. At that time, the bone graft material may not have fully cured, causing insufficient implant osseointegration. At the final observation time, the implants had a survival rate of 66.7%, 10 of the 15 cases. Although the survival rate was low due to repeated failure of implants in certain patients, surviving implants survived for an average of 66.8 ± 51.3 months without any complications except slight marginal bone loss. The risk factors involved in repeated implant failure in this study were sex, location in the maxilla or molar area, and overloading. The implants failed more often in men, which is thought to be due to smoking and excessive occlusal forces. Of the 4 men who smoked, all were long-term smokers for 20 to 50 years at approximately 1 pack a day. Repeated failure of implants occurred numerous times in the maxilla, especially in the maxillary molar area, which is thought to be due to poor bone quality and excessive overloading, as similarly seen in previous studies. Overloading is believed to be the main factor due to repeated intake of hard and tough foods and non-functional parafunctional habits such as bruxism or clenching. There have been many studies in recent years on the association of gene polymorphisms such as IL-1 genotypes as the cause of repetitive implant failures, but no clear association has been proven [23, 24]. Failures were difficult to determine in many of the cases in this study.

A wide variety of 12 implant types was used in this study. TiUniteTM (Nobel Biocare, Brønemark System, Holding AG, Gothenburg, Sweden) is a system that controls roughness by machine cutting the upper aspect of the implant and increasing oxide film through the processing of titanium oxide surfaces as it progresses downward. Osstem® US II (Osstem Implant Co., Busan, Korea) and US III (Osstem Implant Co., Busan, Korea) are submerged type implants that have an external hex connection structure with a sand-blasted surface of alumina and acid etching (SA), with a straight body for US II and a tapered body for US III. SuperLine™ (Dentium, Suwon, Korea) is a double-threaded tapered body designed implant with an internal hex structure and a sand-blasted surface with large grit and acid etching (SLA). Osstem® TS III HA (Osstem Implant Co., Busan, Korea) is a submerged type tapered body implant with an internal hex 11° taper connection and a sand-blasted surface with large grit, acid etching (SLA), and HA coating. 3-I Osseotite® NT (BIOMET 3I, Palm Beach Gardens, FL, USA) is a pure titanium or titanium alloy implant using dual acid etching with hydrochloric acid and sulfuric acid to form the microscopic roughness of the surface. 3-I Nanotite^TM^ (BIOMET 3i, Palm Beach Gardens, FL, USA) possesses calcium phosphate (CaP) crystals of 20–100 nm in size that are dispersed on the surface of OsseoTite, providing it with stronger shear force than typical HA coating. SinusQuick^TM^ IS (Neobiotec, Seoul, Korea) has recently been changed to CMI with a reverse threaded tapered body structure and sandblasting with large grit and an acid etched (SLA) surface. Osstem® TS IV (Osstem Implant Co., Busan, Korea) has a tapered body structure and sand-blasted surface with alumina and acid etching (SA).

In practice, there are many implant failures of unknown cause, and there is a tendency to focus on a specific patient population. Limitations of the retrospective study comprised a small number of cases, lack of a standardized treatment protocols, and no statistical analysis of risk factors. There were also problems with the use of various implant systems, bone grafts, bone graft materials, and barrier membranes and with analysis of evidence based only on medical records and radiographs, making it difficult to estimate the obvious causes of failure. However, systematic prospective clinical research is very difficult, and similar studies are rare. Therefore, this study can help determine basic clinical data and treatment of repeated implant failure of unknown causes.

## Conclusion

Repeated failure of implants in the same site can be caused by overloading, infection, and other unknown causes. Age, sex, and implant sites are estimated to be associated with repeated failure. If appropriate treatment and causes are eliminated, the outcome of re-implantation may be good for repeatedly failed areas. For detailed cause identification and best treatment methods, developmental research and long-term observations are required.

## Additional file

Additional file 1: Case form and result of data. (XLS 186 kb)

## Data Availability

The dataset supporting the conclusions of this article is included within the article and Additional file 1.

## Abbreviations

GBR: Guided bone regeneration
HA: Hydroxy apatite
ISQ: Implant stability quotient
